# Autoimmune conditions and gastric cancer risk in a population-based study in the United Kingdom

**DOI:** 10.1038/s41416-024-02714-7

**Published:** 2024-05-22

**Authors:** John D. Murphy, Shahinaz M. Gadalla, Lesley A. Anderson, Charles S. Rabkin, Chris R. Cardwell, Minkyo Song, M. Constanza Camargo

**Affiliations:** 1 Division of Cancer Epidemiology and Genetics, National Cancer Institute, National Institutes of Health, Bethesda, MD USA; 2 Centre for Health Data Science, Institute of Applied Health Sciences, University of Aberdeen, Aberdeen, UK; 3 School of Medicine, Dentistry and Biomedical Sciences, Queen’s University Belfast, Belfast, UK; 4 Laboratory of Epidemiology and Population Sciences, National Institute on Aging, National Institute of Health, Baltimore, MD USA

**Keywords:** Epidemiology, Gastric cancer, Autoimmune diseases

## Abstract

**Background:**

Although overall incidence of gastric cancer is decreasing, incidence has been increasing among young people in some Western countries. This trend may stem from the increase in autoimmune conditions.

**Methods:**

A nested case-control study of gastric cancer in UK Clinical Practice Research Datalink. Up to ten cancer-free controls were matched to cases by age and sex. Conditional logistic regression was used to calculate odds ratios (OR) and 95% confidence intervals (CI) for associations between analyzable autoimmune conditions (*n* = 34) and gastric cancer with Bonferroni correction. We evaluated associations between pernicious anaemia and other conditions. A meta-analysis of published prospective studies and ours was conducted.

**Results:**

Among 6586 cases (1156 cardia, 1104 non-cardia, and 4334 overlapping/unspecified tumours) and 65,687 controls, any autoimmune condition was associated with gastric cancer (OR = 1.10; 95% CI: 1.01–1.20). Individuals with pernicious anaemia had higher gastric cancer risk than those without (OR = 2.75; 2.19–3.44). Among controls, pernicious anaemia was associated with seven other conditions (OR range: 2.21–29.80). The pooled estimate for any autoimmune condition and gastric cancer was 1.17 (1.14–1.21; *n* = 47,126 cases).

**Conclusion:**

Autoimmunity increases gastric cancer risk. Some autoimmune conditions may be indirectly associated with gastric cancer via pernicious anaemia. Pernicious anaemia could be considered for gastric cancer risk stratification and screening.

## Introduction

Gastric cancer incidence and mortality have been declining in the last few decades [1], trends that parallel the decline in *Helicobacter pylori* infection prevalence [2]. However, data from cancer registries have indicated a recent increase in gastric cancer incidence among younger generations globally, suggesting risk factors other than *H. pylori* are involved [3–5].

A possible explanation for increasing gastric cancer risk is a concomitant rise in the prevalence of autoimmune conditions, especially in high-income countries [6–8]. Autoimmune conditions frequently cause inflammation, which has been found to precede the initiation of many cancers [9, 10]. Gastric cancer, in particular, has been strongly associated with pernicious anaemia [11, 12]. Pernicious anaemia is the result of advanced autoimmune gastritis, a condition involving the destruction of HCl-secreting gastric parietal cells [13–15]. Hypergastrinaemia arising from loss of parietal cells drives development of enterochromaffin cell hyperplasia that can further develop into gastric carcinoids [14, 16]. Also, when parietal cells are lost, less intrinsic factor is produced, and the body’s ability to bind vitamin B12 is diminished [17]. If this gastritis becomes chronic, the resulting exposure to cytokines and other immune system modulators can lead to atrophy. Similar to the well-established cascade of carcinogenesis triggered by chronic *H. pylori* infection [18], autoimmune atrophic gastritis may progress to adenocarcinoma [14].

The “hygiene hypothesis” suggests that more recent generations may lack exposure to microbes such as *H. pylori* early in life due to improved sanitation and increased use of antibiotics and that this absence of exposure may predispose them to allergic and autoimmune conditions later in life [19]. Notably, autoimmunity is associated with gastric adenocarcinoma in addition to carcinoid (i.e., neuroendocrine) tumours [15]. In recent decades, the distribution of gastric cancer incidence has shifted from older male populations toward younger female populations [3, 12, 20]. Autoimmune gastritis disproportionately affects females, which corresponds to the aforementioned trend [21].

Few previous studies have evaluated the association between gastric cancer and multiple autoimmune conditions. The ones that did were limited by the number of exposures they assessed, restricted to older age groups or males, or without consideration of female-specific associations [22–25]. In this longitudinal population-based database, we aimed to examine associations between autoimmune disease and gastric cancer among males and females in a population that includes participants of younger ages and captures exposure at an early age.

## Methods

### Study population

We conducted a nested case-control study within the Clinical Practice Research Datalink (CPRD) GOLD database, a large longitudinal database in the United Kingdom of primary care records linkable to other data sources [26]. Up to ten cancer-free controls, as available, were individually matched to each gastric cancer case on age (single years), sex, general practice, and year of cohort entry. Of 6586 gastric adenocarcinoma cases, only 44 were matched to fewer than ten controls, and only one case was matched to just one control. To ensure that the length of observation was the same within every case-control set, the start of observation for cases and controls was defined as the latest clinic registration date, up-to-standard date, or 1/1/1990 within each case-control set. All observed gastric cancer cases, from the start of data collection in 1987 until the most recent data release in 2017, were included. Incident gastric cancers were ascertained from medical records and/or the cancer registry. Thirty-nine common autoimmune conditions were considered for this study, to be analyzed individually and in the following groupings: systemic/connective tissue (ankylosing spondylitis, Behçet’s disease, polymyalgia rheumatica, polymyositis/dermatomyositis, systemic lupus erythematosus, Reiter’s syndrome, rheumatoid arthritis, sarcoidosis, systemic sclerosis, Sjögren’s syndrome), blood (aplastic anaemia, hemolytic anaemia, pure red cell aplasia, thrombocytopenic purpura), cardiovascular (temporal arteritis, chronic rheumatic heart disease, polyarteritis nodosa, Wegener’s granulomatosis), endocrine (Addison’s disease, type 1 diabetes, Graves’ disease, Hashimoto’s thyroiditis), skin and eye (discoid lupus erythematosus, erythema nodosum, psoriasis, scleritis, localized scleroderma, vitiligo), gastrointestinal including pernicious anaemia (celiac disease, primary sclerosing cholangitis, primary biliary cirrhosis, Crohn’s disease, autoimmune hepatitis, pernicious anaemia, ulcerative colitis), gastrointestinal excluding pernicious anaemia, and nervous system (amyotrophic lateral sclerosis, Guillain-Barré syndrome, multiple sclerosis, myasthenia gravis). Exposure to an autoimmune condition was defined as ever having had a clinical diagnosis of the condition at least two years prior to the index date (i.e., date of matched case’s diagnosis with gastric cancer). The two-year lag between exposure and outcome was implemented to reduce the risk of reverse causation bias. Outcomes and exposures were determined by the International Classification of Diseases, Tenth Revision (ICD-10) code, CPRD MEDCODE, and CPRD Read codes. Smoking status was also determined by CPRD MEDCODE and Read code. A list of MEDCODES and Read codes used in this study is included in Supplementary Tables 1 and 2. Obesity was defined as the last available weight and height recorded at least at age 18 years.

Due to the unequal sex proportion of autoimmune diseases (more frequent in females) and gastric cancer (more frequent in males), we stratified analyses by sex. We also stratified by age at cancer diagnosis (<50 vs. ≥50 years old) because early-onset gastric cancer may have a different etiology and clinical characteristics from cancer arising at an older age [27]. Finally, we stratified the outcome by three anatomical subsites to explore whether their associations with certain autoimmune conditions differed: cardia (ICD-10 code: C160; MEDCODES: 22894, 32022, 37859, 94278, 96094, 97499, 100584), non-cardia (ICD-10 codes: C161-C166; MEDCODES: 19318, 21620, 32362, 41215, 42193, 43572, 48237, 55434, 59092, 65312, 96802), and overlapping/unspecified (ICD-10 codes: C168-C169; MEDCODES: 8386, 10368, 14800, 51690, 55019, 65372).

Many autoimmune conditions co-occur in an individual [28]. To investigate whether a specific autoimmune condition association with gastric cancer was independent or co-occurred with more direct pathologic changes occurring in pernicious anaemia, we assessed the association between autoimmune conditions and pernicious anaemia.

We conducted four sensitivity analyses: (i) analyses of the associations between gastric cancer and autoimmune conditions adjusted for pernicious anaemia; (ii) a complete case analysis of the association between autoimmune conditions and gastric cancer, owing to a relatively large proportion of missing data on covariates including smoking status (28.4%) and body mass index (BMI; 35.4%); (iii) an analysis defining exposure as having had at least two diagnoses of a given autoimmune condition, to ensure that the condition was not misdiagnosed the first time; and (iv) an analysis with a five-year lag between pernicious anaemia diagnosis and gastric cancer diagnosis to minimize reverse causation.

Finally, we also undertook a fixed-effect meta-analysis of large prospective cohorts in which associations between at least five specific autoimmune conditions and gastric cancer had been made [22–25]. Heterogeneity in the meta-analysis was tested via *I*^*2*^ and Cochrane’s *Q*, for which *p* values were reported.

### Statistical analysis

Conditional logistic regression was used to calculate odds ratios (OR) and 95% confidence intervals (CI) for the association between autoimmune conditions (all together, individually, and grouped by organ site) and incident gastric cancer. ORs were adjusted for age, sex, BMI (< 20, 20–24, 25–29, 30–39, ≥40 kg/m^2^, missing), and smoking status (never, former, current, unspecified). Adjusting for matching factors can be important in case the matching itself introduces a relationship between the confounder and the exposure that would not otherwise have been present [29]. Five autoimmune conditions (hemolytic anaemia, pure red cell aplasia, polyarteritis nodosa, primary sclerosing cholangitis, and amyotrophic lateral sclerosis) were not analyzable as individual conditions for the association with gastric cancer due to having zero observations among cases but were included as part of the analysis of exposure to any autoimmune condition. A Bonferroni correction to account for multiple hypothesis testing (for each of the analyzable autoimmune conditions) was applied to the regression model results based on the number of analyzable autoimmune conditions (*n* = 34; *p*_*Bonferroni*_ = 0.05/34 = 0.00147). ORs with *p*-values <0.05 prior to, but not significant at Bonferroni corrected level were considered nominally significant. Heterogeneity by sex, age at cancer diagnosis (<50 *vs*. ≥50 years), and anatomical subsite (cardia, non-cardia, overlapping/unspecified) was separately tested via a Wald test at the 95% confidence level using interaction terms in the logistic regression models [29]. In addition, we estimated associations between pernicious anaemia and autoimmune conditions via logistic regression among controls. All analyses were conducted in R statistical software (version 4.1.2) except for the fixed-effect meta-analyses of previous studies of autoimmunity and gastric cancer as well as pernicious anaemia, for which we used Stata version 17.

### Patient and public involvement

Patients and the public were not explicitly involved in the conception or design of this study.

## Results

Females comprised 37% of cases and controls (Table 1). The median age for both cases and controls was 75 years (IQR, 67–82). 11.5% of cases (*n* = 758) had been diagnosed with any autoimmune condition, compared with 10.2% of controls (*n* = 6670). Co-occurrence of conditions (i.e., two or more) was more common in cases than controls (1.5% vs. 1.0%; *p* < 0.001). Cases were more likely than controls to be current smokers (17.3% vs. 13.5%; *p* < 0.001) but less likely to be overweight ( ≥25 kg/m^2^; 36.5% vs. 42.1%; *p* < 0.001) and obese ( ≥30 kg/m^2^; 12.8% vs. 15.4%; *p* < 0.001). The median follow-up time for the whole data set was 11.2 years (IQR: 6.6–14.7).

**Table 1:** Descriptive characteristics of gastric cancer cases and matched cancer-free controls in the Clinical Research Practice Datalink (*n* = 72,273).

	Case (N = 6,586)	Control (*N* = 65,687)	*p*
	*N* (%)	*N* (%)	
**Female**	2451 (37.2)	24,442 (37.2)	N/A^a^
**Age in years, median (IQR)**	75 (67–82)	75 (67–82)	N/A^a^
**Age category**			
**<40**	63 (1.0)	633 (1.0)	
**40–49**	214 (3.2)	2164 (3.3)	
**50–59**	524 (8.0)	5240 (8.0)	
**60–69**	1288 (19.6)	12,896 (19.6)	
**≥70**	4497 (68.3)	44,754 (68.1)	
**Number of autoimmune condition diagnoses**			< 0.001
**None**	5828 (88.5)	59,017 (89.8)	
**Any**	758 (11.5)	6670 (10.2)	
**1**	658 (10.0)	6052 (9.2)	
**2**	91 (1.4)	568 (0.9)	
**≥3**	9 (0.1)	50 (0.1)	
**Smoking status**			< 0.001
**Current**	1141 (17.3)	8845 (13.5)	
**Never**	1282 (19.5)	16,602 (25.3)	
**Former**	2223 (33.8)	22,273 (33.9)	
**Missing**	1940 (29.5)	17,967 (27.4)	
**Body mass index (kg/m**^**2**^**)**			< 0.001
**<20**	446 (6.8)	2064 (3.1)	
**20–24**	1386 (21.0)	13,086 (19.9)	
**25–29**	1563 (23.7)	17,364 (26.4)	
**30–39**	790 (12.0)	9410 (14.3)	
**≥40**	55 (0.8)	692 (1.1)	
**Missing**	2346 (35.6)	23,071 (35.1)	
**Alcohol status**			< 0.001
**Never**	508 (7.7)	4901 (7.5)	
**Former**	484 (7.3)	4248 (6.5)	
**Current**	2815 (42.7)	30,282 (46.1)	
**Missing**	2779 (42.2)	26,256 (40.0)	

^a^Matching variables (therefore, no statistical test performed).

### Autoimmune conditions and gastric cancer

Having ever had any autoimmune condition was nominally associated with increased odds of gastric cancer (OR = 1.10, 95% CI: 1.01–1.20; Table 2). However, this association was attenuated when pernicious anaemia was excluded (OR = 1.00, 95% CI: 0.92–1.10). In the disease group analysis, only gastrointestinal conditions, including pernicious anaemia, were significantly associated with gastric cancer (OR = 1.68, 95% CI: 1.40–2.00), which also attenuated when excluding pernicious anaemia (OR = 0.92, 95% CI: 0.69–1.23). Pernicious anaemia was significantly associated with gastric cancer (OR = 2.75, 95% CI: 2.19–3.44). Aplastic anaemia was nominally associated (*p* < 0.05): (OR = 14.00, 95% CI: 2.33–84.20).

**Table 2:** Association between autoimmune conditions and gastric cancer in the Clinical Practice Research Datalink (*n* = 72,273).

Autoimmune condition	Cases (*N* = 6586)	Controls (*N* = 65,687)	OR (95% CI)	*p*
	*N* (%)	*N* (%)		
Any autoimmune condition	634 (9.6)	5788 (8.8)	1.10 (1.01–1.20)	0.03*
Any autoimmune condition, excluding pernicious anaemia	554 (8.4)	5484 (8.3)	1.00 (0.92–1.10)	0.94
Systemic/connective tissue	206 (3.1)	1973 (3.0)	1.04 (0.89–1.20)	0.65
Ankylosing spondylitis	8 (0.1)	89 (0.1)	0.90 (0.44–1.86)	0.77
Behçet’s disease	^	7 (0.0)	1.35 (0.17–11.00)	0.78
Polymyalgia rheumatica	99 (1.5)	967 (1.5)	1.01 (0.82–1.25)	0.92
Polymyositis/Dermatomyositis	^	14 (0.0)	0.72 (0.10–5.50)	0.75
Systemic lupus erythematosus	^	35 (0.1)	0.56 (0.14–2.35)	0.43
Reiter’s syndrome	^	28 (0.0)	1.05 (0.32–3.46)	0.93
Rheumatoid arthritis	95 (1.4)	802 (1.2)	1.18 (0.95–1.46)	0.14
Sarcoidosis	6 (0.1)	76 (0.1)	0.78 (0.34–1.80)	0.57
Systemic sclerosis	^	14 (0.0)	2.08 (0.60–7.25)	0.25
Sjögren’s syndrome	6 (0.1)	50 (0.1)	1.16 (0.49–2.70)	0.74
Blood	^	46 (0.1)	1.10 (0.44–2.79)	0.83
Aplastic anaemia	^	^	14.00 (2.33–84.20)	< 0.01*
Hemolytic anaemia	^	^	N/A	N/A
Pure red cell aplasia	^	^	N/A	N/A
Thrombocytopenic purpura	^	41 (0.1)	0.50 (0.12–2.08)	0.34
Cardiovascular	36 (0.5)	383 (0.6)	0.94 (0.67–1.32)	0.71
Temporal arteritis	16 (0.2)	213 (0.3)	0.75 (0.45–1.24)	0.26
Chronic rheumatic heart disease	19 (0.3)	158 (0.2)	1.21 (0.75–1.95)	0.44
Polyarteritis nodosa	^	8 (0.0)	N/A	N/A
Wegener’s granulomatosis	^	7 (0.0)	1.43 (0.18–11.60)	0.74
Endocrine	19 (0.3)	240 (0.4)	0.79 (0.49–1.26)	0.32
Addison’s disease	^	22 (0.0)	1.42 (0.42–4.75)	0.57
Type 1 diabetes	12 (0.2)	166 (0.3)	0.71 (0.40–1.28)	0.26
Graves’ disease	^	30 (0.0)	1.33 (0.47–3.79)	0.59
Hashimoto’s thyroiditis	^	23 (0.0)	0.45 (0.06–3.30)	0.43
Skin and eye	260 (3.9)	2451 (3.7)	1.06 (0.93–1.21)	0.39
Discoid lupus erythematosus	^	25 (0.0)	1.56 (0.54–4.49)	0.41
Erythema nodosum	10 (0.2)	61 (0.1)	1.63 (0.83–3.19)	0.15
Psoriasis	213 (3.2)	2050 (3.1)	1.04 (0.90–1.20)	0.61
Scleritis	20 (0.3)	228 (0.3)	0.86 (0.55–1.37)	0.53
Localized scleroderma	^	12 (0.0)	1.62 (0.36–7.26)	0.53
Vitiligo	11 (0.2)	99 (0.2)	1.10 (0.59–2.04)	0.77
Gastrointestinal including pernicious anaemia	148 (2.2)	883 (1.3)	1.68 (1.40–2.00)	<0.001**
Celiac disease	12 (0.2)	119 (0.2)	0.98 (0.54–1.77)	0.95
Primary sclerosing cholangitis	^	^	N/A	N/A
Primary biliary cirrhosis	^	22 (0.0)	0.91 (0.21–3.85)	0.89
Crohn’s disease	16 (0.2)	134 (0.2)	1.17 (0.69–1.96)	0.57
Autoimmune hepatitis	^	6 (0.0)	1.65 (0.20–13.70)	0.64
Pernicious anaemia	99 (1.5)	363 (0.6)	2.75 (2.19–3.44)	<0.001**
Ulcerative colitis	25 (0.4)	275 (0.4)	0.91 (0.60–1.37)	0.64
Gastrointestinal excluding pernicious anaemia	50 (0.8)	535 (0.8)	0.92 (0.69–1.23)	0.58
Nervous	16 (0.2)	171 (0.3)	0.95 (0.57–1.58)	0.83
Amyotrophic lateral sclerosis	^	^	N/A	N/A
Guillain-Barré syndrome	^	28 (0.0)	0.70 (0.17–2.92)	0.62
Multiple sclerosis	9 (0.1)	111 (0.2)	0.83 (0.42–1.64)	0.59
Myasthenia gravis	^	33 (0.1)	1.52 (0.59–3.90)	0.38

Odds ratios (OR) and 95% confidence intervals (CI) calculated using conditional logistic regression adjusted for age (in years), sex (binary), body mass index (< 20, 20–24, 25–29, 30–39, ≥ 40 kg/m^2^, unspecified), and smoking status (never, former, current, unspecified).

N/A labels are for ORs that were inestimable due to lack of observations.

To reduce the probability of reverse causation, individuals with an autoimmune condition diagnosis less than two years prior to index were considered unexposed.

*p-value is nominally statistically significant at a Type I error rate of 0.05.

**p-value is statistically significant with a Bonferroni correction factor of 34 (i.e., at an uncorrected Type I error rate of 0.00147).

Individuals with more than one autoimmune condition diagnosis were only counted as one observation in grouped variables such as “Any autoimmune condition.” Therefore, the sum of cases with an autoimmune condition diagnosis in the whole table will appear greater than the number of cases considered exposed to a group variable.

Cells with “^” represent counts of ≤5, which have been suppressed according to CPRD privacy standards.

### Autoimmune conditions and gastric cancer by sex and age

The association between any autoimmune condition and gastric cancer was similar for both sexes, as was the magnitude of association between pernicious anaemia and gastric cancer (Table 3). Associations were also similar by age group. No associations were significant among the younger age group. Among older individuals, significant findings were observed for pernicious anaemia (OR = 2.74, 95% CI: 2.18–3.43), but no evidence of a difference was observed between the older and younger groups (*p*_*heterogeneity*_ = 0.62), and the point estimate was greater among younger individuals (OR = 4.30, 95% CI: 0.39–47.70).

**Table 3:** Associations between autoimmune conditions and gastric cancer in the Clinical Practice Research Datalink, stratified by sex and age (n = 72,273)

	Any autoimmune condition				Any autoimmune condition excluding pernicious anaemia				Pernicious anaemia			
*Strata*	Among Cases, *N* (%)	OR (95% CI)	*p*	*p*_*heterogeneity*_	Among Cases, N (%)	OR (95% CI)	*p*	*p*_*heterogeneity*_	Among Cases, N (%)	OR (95% CI)	*p*	*p*_*heterogeneity*_
Sex												
Male	415 (10.0)	1.08 (0.96–1.21)	0.21	0.60	366 (8.9)	0.99 (0.88–1.12)	0.87	0.74	64 (1.5)	2.66 (1.95–3.62)	<0.001**	0.79
Female	281 (11.5)	1.13 (0.99–1.29)	0.07		244 (10.0)	1.02 (0.89–1.18)	0.75		47 (1.9)	2.86 (2.05–3.97)	<0.001**	
Age												
<50 years	14 (5.1)	0.92 (0.52–1.62)	0.77	0.58	14 (5.1)	0.93 (0.53–1.65)	0.81	0.85	^	4.30 (0.39–47.70)	0.23	0.62
≥50 years	620 (9.8)	1.10 (1.01–1.21)	0.03*		540 (8.6)	1.01 (0.92–1.10)	0.9		98 (1.6)	2.74 (2.18–3.43)	<0.001**	

Odds ratios (OR) and 95% confidence intervals (CI) calculated using conditional logistic regression adjusted for age (in years), sex (binary), body mass index < 20, 20–24, 25–29, 30–39, ≥ 40 kg/m^2^, unspecified), and smoking status (never, former, current, unspecified).

N/A labels are for ORs that were inestimable due to lack of observations.

To reduce the probability of reverse causation, individuals with an autoimmune condition diagnosis less than two years prior to index were considered unexposed.

*p-value is statistically significant at a Type I error rate of 0.05.

**p-value is statistically significant with a Bonferroni correction factor of 34 (i.e., at an uncorrected Type I error rate of 0.00147).

Autoimmune condition groupings comprise: systemic/connective tissue (ankylosing spondylitis, Behçet’s disease, polymyalgia rheumatica, polymyositis/dermatomyositis, systemic lupus erythematosus, Reiter’s syndrome, rheumatoid arthritis, sarcoidosis, systemic sclerosis, Sjögren’s syndrome), blood (aplastic anaemia, hemolytic anaemia, pure red cell aplasia, thrombocytopenic purpura), cardiovascular (temporal arteritis, chronic rheumatic heart disease, polyarteritis nodosa, Wegener’s granulomatosis), endocrine (Addison’s disease, type 1 diabetes, Graves’ disease, Hashimoto’s thyroiditis), skin and eye (discoid lupus erythematosus, erythema nodosum, psoriasis, scleritis, localized scleroderma, vitiligo), gastrointestinal including pernicious anaemia (celiac disease, primary sclerosing cholangitis, primary biliary cirrhosis, Crohn’s disease, autoimmune hepatitis, pernicious anaemia, ulcerative colitis), gastrointestinal excluding pernicious anaemia, nervous system (amyotrophic lateral sclerosis, Guillain-Barré syndrome, multiple sclerosis, myasthenia gravis).

Individuals with more than one autoimmune condition diagnosis were only counted as one observation in grouped variables such as “Any autoimmune condition.” Therefore, the sum of cases with an autoimmune condition diagnosis in the whole table will appear greater than the number of cases considered exposed to a group variable.

Cells with “^” represent counts of ≤ 5, which have been suppressed according to CPRD privacy standards.

### Autoimmune conditions and anatomical subsites of gastric cancer

Among gastric cancer cases, 17.6% (*n* = 1152) were cardia, 16.8% (*n* = 1100) were non-cardia tumours, and 65.8% (*n* = 4334) were overlapping (*n* = 71) or unspecified (*n* = 4263) tumours (Table 4). Associations with any autoimmune condition were statistically significant for non-cardia tumours but not for cardia tumours (*p*_*heterogeneity*_ = 0.10). Pernicious anaemia was significantly associated with non-cardia gastric cancer (OR = 3.91; 95% CI, 2.31–6.62) but not with cardia cancer (OR = 1.71, 95% CI: 0.80–3.65; *p*_*heterogeneity*_ = 0.07). Overlapping/unspecified tumours were also significantly associated with pernicious anaemia (OR = 2.70, 95% CI: 2.07–3.53) and nominally associated with aplastic anaemia (OR = 9.82, 95% CI: 1.38–70.00).

**Table 4:** Associations between autoimmune conditions and different gastric cancer anatomical subsites (*n* = 72,273).

Autoimmune condition	Cardia (*N* = 1152)		Non-cardia (*N* = 1100)		Overlapping/Unspecified (*N* = 4334)		*P*_*heterogeneity*_ Cardia vs. Non-cardia
	*N* (%)	OR (95% CI)	*N* (%)	OR (95% CI)	*N* (%)	OR (95% CI)	
Any autoimmune condition	102 (8.8)	1.05 (0.85–1.30)	121 (11.0)	1.23 (1.00–1.50)*	411 (9.5)	1.08 (0.97–1.20)	0.36
Any autoimmune condition excluding pernicious anaemia	96 (8.3)	1.03 (0.82–1.28)	103 (9.3)	1.08 (0.87–1.33)	355 (8.2)	0.98 (0.87–1.10)	0.83
Systemic/connective tissue	35 (3.0)	1.22 (0.85–1.74)	39 (3.5)	1.10 (0.79–1.55)	132 (3.0)	0.98 (0.81–1.17)	0.99
Ankylosing spondylitis	^	2.16 (0.61–7.62)	^	0.66 (0.09–4.96)	^	0.67 (0.24–1.85)	0.36
Behçet’s disease	^	N/A	^	N/A	^	1.57 (0.19–13.00)	N/A
Polymyalgia rheumatica	16 (1.4)	1.20 (0.71–2.04)	19 (1.7)	1.04 (0.64–1.68)	64 (1.5)	0.97 (0.74–1.26)	0.84
Polymyositis/Dermatomyositis	^	3.34 (0.35–32.10)	^	N/A	^	N/A	N/A
Systemic lupus erythematosus	^	N/A	^	N/A	^	0.71 (0.17–3.00)	N/A
Reiter’s syndrome	^	1.95 (0.23–16.70)	^	N/A	^	0.94 (0.22–4.03)	N/A
Rheumatoid arthritis	14 (1.2)	1.19 (0.68–2.09)	19 (1.7)	1.30 (0.81–2.11)	62 (1.4)	1.13 (0.87–1.48)	0.41
Sarcoidosis	^	2.15 (0.62–7.47)	^	0.96 (0.12–7.47)	^	0.38 (0.09–1.57)	0.43
Systemic sclerosis	^	9.66 (0.60–155.00)	^	N/A	^	1.76 (0.39–7.97)	0.97
Sjögren’s syndrome	^	2.04 (0.44–9.53)	^	N/A	^	1.19 (0.42–3.39)	0.95
Blood	^	2.82 (0.59–13.60)	^	0.79 (0.10–6.09)	^	0.79 (0.19–3.35)	0.62
Aplastic anaemia	^	N/A	^	N/A	^	9.82 (1.38–70.00)*	N/A
Hemolytic anaemia	^	N/A	^	N/A	^	N/A	N/A
Pure red cell aplasia	^	N/A	^	N/A	0 (0.0)	N/A	N/A
Thrombocytopenic purpura	^	1.67 (0.20–13.90)	^	0.94 (0.12–7.43)	^	N/A	0.92
Cardiovascular	^	0.54 (0.17–1.71)	9 (0.8)	1.29 (0.64–2.59)	24 (0.6)	0.93 (0.61–1.42)	0.09
Temporal arteritis	^	0.72 (0.17–3.03)	^	0.85 (0.30–2.36)	10 (0.2)	0.72 (0.38–1.36)	0.51
Chronic rheumatic heart disease	^	0.37 (0.05–2.73)	^	2.48 (0.93–6.61)	13 (0.3)	1.18 (0.66–2.10)	0.09
Polyarteritis nodosa	^	N/A	^	N/A	^	N/A	N/A
Wegener’s granulomatosis	^	N/A	^	N/A	^	1.63 (0.20–13.50)	N/A
Endocrine	^	0.92 (0.33–2.57)	^	1.03 (0.37–2.88)	11 (0.3)	0.70 (0.38–1.28)	0.92
Addison’s disease	^	N/A	^	N/A	^	1.76 (0.52–6.00)	N/A
Type 1 diabetes	^	0.80 (0.25–2.60)	^	0.65 (0.16–2.72)	7 (0.2)	0.70 (0.33–1.52)	0.91
Graves’ disease		5.06 (0.46–55.80)	^	1.69 (0.20–14.00)	^	0.90 (0.21–3.85)	0.82
Hashimoto’s thyroiditis	^	N/A	^	N/A	^	N/A	N/A
Skin and eye	45 (3.9)	1.02 (0.74–1.39)	47 (4.3)	1.11 (0.81–1.51)	168 (3.9)	1.05 (0.90–1.24)	0.83
Discoid lupus erythematosus	^	N/A	^	N/A	^	1.96 (0.67–5.75)	N/A
Erythema nodosum	^	N/A	^	2.89 (0.60–13.90)	8 (0.2)	1.77 (0.83–3.77)	0.96
Psoriasis	37 (3.2)	1.01 (0.72–1.43)	35 (3.2)	0.97 (0.68–1.37)	141 (3.3)	1.06 (0.89–1.26)	0.95
Scleritis	^	0.59 (0.18–1.89)	7 (0.6)	1.98 (0.87–4.52)	10 (0.2)	0.69 (0.36–1.31)	0.31
Localized scleroderma	^	9.66 (0.60–155.00)	^	N/A	^	1.08 (0.14–8.56)	N/A
Vitiligo	^	2.47 (0.83–7.40)	^	1.42 (0.43–4.78)	^	0.64 (0.23–1.75)	0.62
Gastrointestinal including pernicious anaemia	13 (1.1)	0.82 (0.46–1.45)	27 (2.4)	1.87 (1.23–2.83)*	108 (2.5)	1.86 (1.51–2.30)**	0.10
Celiac disease	^	1.15 (0.27–4.98)	^	1.22 (0.37–4.07)	7 (0.2)	0.87 (0.40–1.89)	0.97
Primary sclerosing cholangitis	^	N/A	^	N/A	^	N/A	N/A
Primary biliary cirrhosis	^	N/A	^	N/A	^	1.44 (0.33–6.34)	N/A
Crohn’s disease	^	0.31 (0.04–2.25)	^	1.33 (0.40–4.49)	12 (0.3)	1.47 (0.80–2.69)	0.34
Autoimmune hepatitis	^	N/A	^	N/A	^	2.43 (0.27–21.80)	N/A
Pernicious anaemia	8 (0.7)	1.71 (0.80–3.65)	20 (1.8)	3.91 (2.31–6.62)**	71 (1.6)	2.70 (2.07–3.53)**	0.07
Ulcerative colitis	^	0.47 (0.15–1.50)	^	0.64 (0.20–2.07)	19 (0.4)	1.15 (0.72–1.86)	0.84
Gastrointestinal excluding pernicious anaemia	^	0.44 (0.18–1.09)	8 (0.7)	0.81 (0.39–1.67)	37 (0.9)	1.12 (0.79–1.57)	0.64
Nervous	^	1.20 (0.36–3.99)	^	0.94 (0.29–3.06)	10 (0.2)	0.89 (0.47–1.71)	1.00
Amyotrophic lateral sclerosis	^	N/A	^	N/A	^	N/A	N/A
Guillain-Barré syndrome	^	N/A	^	N/A	^	0.98 (0.23–4.17)	N/A
Multiple sclerosis	^	1.06 (0.25–4.55)	^	0.91 (0.21–3.89)	^	0.74 (0.30–1.84)	0.93
Myasthenia gravis	^	5.12 (0.46–56.50)	^	1.70 (0.20–14.10)	^	1.20 (0.36–3.97)	0.91

Odds ratios (OR) and 95% confidence intervals (CI) calculated using conditional logistic regression adjusted for age (in years), sex (binary), body mass index ( < 20, 20–24, 25–29, 30–39, ≥ 40 kg/m^2^, unspecified), and smoking status (never, former, current, unspecified).

N/A labels are for ORs that were inestimable due to lack of observations.

To reduce the probability of reverse causation, individuals with an autoimmune condition diagnosis less than two years prior to index were considered unexposed.

*p-value is nominally statistically significant at a Type I error rate of 0.05

**p-value is statistically significant with a Bonferroni correction factor of 34 (i.e., at an uncorrected Type I error rate of 0.00147).

Autoimmune condition groupings comprise: systemic/connective tissue (ankylosing spondylitis, Behçet’s disease, polymyalgia rheumatica, polymyositis/dermatomyositis, systemic lupus erythematosus, Reiter’s syndrome, rheumatoid arthritis, sarcoidosis, systemic sclerosis, Sjögren’s syndrome), blood (aplastic anaemia, hemolytic anaemia, pure red cell aplasia, thrombocytopenic purpura), cardiovascular (temporal arteritis, chronic rheumatic heart disease, polyarteritis nodosa, Wegener’s granulomatosis), endocrine (Addison’s disease, type 1 diabetes, Graves’ disease, Hashimoto’s thyroiditis), skin and eye (discoid lupus erythematosus, erythema nodosum, psoriasis, scleritis, localized scleroderma, vitiligo), gastrointestinal including pernicious anaemia (celiac disease, primary sclerosing cholangitis, primary biliary cirrhosis, Crohn’s disease, autoimmune hepatitis, pernicious anaemia, ulcerative colitis), gastrointestinal excluding pernicious anaemia, nervous system (amyotrophic lateral sclerosis, Guillain-Barré syndrome, multiple sclerosis, myasthenia gravis).

Individuals with more than one autoimmune condition diagnosis were only counted as one observation in grouped variables such as “Any autoimmune condition.” Therefore, the sum of cases with an autoimmune condition diagnosis in the whole table will appear greater than the number of cases considered exposed to a group variable.

Cells with “^” represent counts of ≤ 5, which have been suppressed according to CPRD privacy standards.

### Sensitivity analyses

When adjusted for pernicious anaemia, the associations between gastric cancer and aplastic anaemia (OR = 14.00, 95% CI: 2.33–84.30) remained nominally significant (Supplementary Table 3).

In the complete-case analysis (*N* = 44,762; 62% of the entire study population), any autoimmune condition was nominally associated with gastric cancer (OR = 1.17, 95% CI: 1.06–1.29; Supplementary Table 4) with similar magnitude of association using the entire analytical set (OR = 1.10, 95% CI: 1.01–1.20; Table 2). Pernicious anaemia was also significantly associated with gastric cancer (OR = 2.62, 95% CI: 2.05–3.35). Other gastrointestinal autoimmune conditions were not significantly associated with gastric cancer. Having had any systemic/connective tissue autoimmune condition was also nominally associated with gastric cancer (OR = 1.20, 95% CI: 1.02–1.40). Among these, rheumatoid arthritis was nominally associated with gastric cancer (OR = 1.36, 95% CI: 1.08–1.72).

The association between pernicious anaemia and gastric cancer was stronger when restricted to individuals with at least two diagnoses of the same autoimmune condition (OR = 3.78; 2.46–5.81; Supplementary Table 5). Nominal associations with gastric cancer were observed for erythema nodosum (OR = 4.38, 95% CI: 1.13–17.00) and Reiter’s syndrome (OR = 18.40, 95% CI: 1.67–204.00).

When a five-year lag between pernicious anaemia diagnosis and gastric cancer diagnosis was considered instead of a two-year lag, the association remained statistically significant (OR, 2.79; 95% CI, 2.14–3.65).

### Meta-analysis of Published Literature of Prospective Studies on Autoimmune Diseases and Gastric Cancer

After meta-analyzing the risk estimates from the current CPRD and previously published SEER-Medicare, the Swedish cohort, and the Veterans Affairs’ cohort, including 46,716 cases, the association for any autoimmune condition with gastric cancer risk was OR_meta_ = 1.17 (95% CI: 1.14–1.21; Supplementary Table 6). The pooled association for pernicious anaemia with any subsite was OR_meta_ = 2.05 (95% CI: 1.92–2.17). Cardia tumours were also positively associated with pernicious anaemia (OR_meta_ = 1.28, 95% CI: 1.10–1.46), although less strongly than non-cardia tumours (OR_meta_ = 2.04, 95% CI: 1.87–2.22). Graves’ disease was significantly positively associated with any gastric cancer (OR_meta_ = 1.28, 95% CI: 1.11–1.45) and with non-cardia tumours specifically (OR_meta_ = 1.48, 95% CI: 1.14–1.83). Having any gastric tumour was also positively associated with primary biliary cirrhosis (OR_meta_ = 1.66, 95% CI: 1.35–1.97) and myasthenia gravis (OR_meta_ = 1.26, 95% CI: 1.06–1.47). Multiple sclerosis was significantly inversely associated with gastric cancer (OR_meta_ = 0.73, 95% CI: 0.56–0.90), as was celiac disease (OR_meta_ = 0.65, 95% CI: 0.44–0.87) and temporal arteritis (OR = 0.80, 95% CI: 0.63–0.97).

### Autoimmune conditions and pernicious anaemia among controls

Eighteen percent of controls with pernicious anaemia had one or more other autoimmune conditions. Two disease groups were positively associated with pernicious anaemia –systemic/connective tissue conditions (OR = 1.90, 95% CI: 1.31–2.67) and gastrointestinal conditions (OR = 4.41, 95% CI: 2.71–6.77; Table 5). Seven individual autoimmune conditions were associated with pernicious anaemia at Bonferroni significant level: hemolytic anaemia (OR = 29.80, 95% CI: 1.51–206.00), Crohn’s disease (OR = 7.09, 95% CI: 3.17–13.70), vitiligo (OR = 7.09, 95% CI: 2.76–14.90), Sjögren’s disease (OR = 6.76, 95% CI: 1.64–18.50), celiac disease (OR = 6.40, 95% CI: 2.69–12.80), type 1 diabetes (OR = 3.78, 95% CI: 1.48–7.85), and rheumatoid arthritis (OR = 2.21, 95% CI: 1.26–3.58).

**Table 5:** Association between autoimmune conditions and pernicious anaemia in controls in the Clinical Practice Research Datalink (n = 65,687).

Autoimmune Condition	Among Pernicious Anaemia Patients, *N* (%)	OR (95% CI)^a^	*p*
Any autoimmune condition excluding pernicious anaemia	85 (18.2)	1.98 (1.55–2.49)	<0.001**
Systemic/connective tissue	33 (7.1)	1.90 (1.31–2.67)	<0.001**
Ankylosing spondylitis	^	2.83 (0.47–9.01)	0.15
Behçet’s disease	^	N/A	N/A
Polymyalgia rheumatica	15 (3.2)	1.60 (0.92–2.59)	0.07
Polymyositis/Dermatomyositis	^	N/A	N/A
Systemic lupus erythematosus	^	N/A	N/A
Reiter’s syndrome	^	N/A	N/A
Rheumatoid arthritis	15 (3.2)	2.21 (1.26–3.58)	0.003*
Sarcoidosis	^	N/A	N/A
Systemic sclerosis	^	N/A	N/A
Sjögren’s syndrome	^	6.76 (1.64–18.50)	0.001**
Blood	^	4.90 (0.80–15.80)	0.03*
Aplastic anaemia	^	N/A	N/A
Hemolytic anaemia	^	29.80 (1.51–206.00)	0.003*
Pure red cell aplasia	^	N/A	N/A
Thrombocytopenic purpura	^	2.85 (0.16–13.10)	0.30
Cardiovascular	^	0.61 (0.10–1.91)	0.49
Temporal arteritis	^	0.50 (0.03–2.24)	0.49
Chronic rheumatic heart disease	^	0.86 (0.05–3.85)	0.88
Polyarteritis nodosa	^	N/A	N/A
Wegener’s granulomatosis	^	N/A	N/A
Endocrine	6 (1.3)	2.71 (1.06–5.59)	0.02*
Addison’s disease	^	N/A	N/A
Type 1 diabetes	6 (1.3)	3.78 (1.48–7.85)	0.001**
Graves’ disease	^	N/A	N/A
Hashimoto’s thyroiditis	^	N/A	N/A
Skin and eye	29 (6.2)	1.45 (0.97–2.07)	0.06
Discoid lupus erythematosus	^	N/A	N/A
Erythema nodosum	^	2.08 (0.12–9.46)	0.47
Psoriasis	20 (4.3)	1.18 (0.73–1.80)	0.47
Scleritis	^	1.02 (0.17–3.18)	0.98
Localized scleroderma	^	N/A	N/A
Vitiligo	6 (1.3)	7.09 (2.76–14.90)	<0.0001**
Gastrointestinal excluding pernicious anaemia	20 (4.3)	4.41 (2.71–6.77)	<0.0001**
Celiac disease	7 (1.5)	6.40 (2.69–12.80)	<0.0001**
Primary sclerosing cholangitis	^	N/A	N/A
Primary biliary cirrhosis	^	N/A	N/A
Crohn’s disease	8 (1.7)	7.09 (3.17–13.70)	<0.0001**
Autoimmune hepatitis	^	17.50 (0.93–99.08)	0.51
Ulcerative colitis	^	2.15 (0.76–4.69)	0.09
Nervous	^	1.42 (0.24–4.46)	0.62
Amyotrophic lateral sclerosis	^	N/A	N/A
Guillain-Barré syndrome	^	N/A	N/A
Multiple sclerosis	^	1.13 (0.06–5.07)	0.90
Myasthenia gravis	^	3.53 (0.20–16.30)	0.21

Odds ratios (OR) and 95% confidence intervals (CI) calculated using conditional logistic regression adjusted for age (in years), sex (binary), body mass index ( <20, 20–24, 25–29, 30–39, ≥40 kg/m^2^, unspecified), and smoking status (never, former, current, unspecified).

N/A labels are for ORs that were inestimable due to lack of observations.

*p-value is nominally statistically significant at a Type I error rate of 0.05

**p-value is statistically significant with a Bonferroni correction factor of 34 (i.e., at an uncorrected Type I error rate of 0.00147).

Individuals with more than one autoimmune condition diagnosis were only counted as one observation in grouped variables such as “Any autoimmune condition.” Therefore, the sum of cases with an autoimmune condition diagnosis in the whole table will appear greater than the number of cases considered exposed to a group variable.

Cells with “^” represent counts of ≤ 5, which have been suppressed according to CPRD privacy standards.

In a meta-analysis of our CPRD findings with those of our previous SEER-Medicare study (Supplementary Table 7), five autoimmune conditions were significantly associated with pernicious anaemia: Crohn’s disease (OR_meta_ = 3.84, 95% CI: 2.50–5.17), celiac disease (OR_meta_ = 3.00, 95% CI: 1.21–4.80), rheumatoid arthritis (OR_meta_ = 1.77, 95% CI: 1.53–2.00), ulcerative colitis (OR_meta_ = 1.62, 95% CI: 1.03–2.21), and polymyalgia rheumatica (OR_meta_ = 1.33, 95% CI: 1.01–1.65) [24].

## Discussion

In this large nested case-control study, we confirmed a positive association between pernicious anaemia and gastric cancer and, additionally, expanded on the associations between pernicious anaemia and other autoimmune conditions. Although not statistically significant after Bonferroni correction, we observed a suggestive positive gastric cancer association with aplastic anaemia. Autoimmune conditions positively associated with pernicious anaemia included type 1 diabetes, hemolytic anaemia, Crohn’s disease, celiac disease, Sjögren’s disease, rheumatoid arthritis, and vitiligo. These results imply the role of autoimmune conditions in inflammation, which involves increased cell proliferation and DNA damage, both of which contribute to carcinogenesis [22, 30, 31].

Our study replicated the association between pernicious anaemia and gastric cancer established in previous research [12, 17, 23, 24]. Pernicious anaemia results from the gradual loss of gastric parietal cells via autoimmune destruction, hampering the ability to absorb vitamin B12 [15, 22, 23]. In the course of this atrophic gastritis, chronic inflammation may increase the risk of gastric cancer [9, 10]. In accordance with the distribution of the parietal cells in the stomach, pernicious anaemia has a stronger association with non-cardia gastric cancer [32]. Pernicious anaemia was not associated with gastric cardia cancer in our study but, when combined with a previous study of elderly adults in the United States, the pooled estimate was significant, although the magnitude of association was smaller than in non-cardia gastric cancer [24]. Some non-cardia tumours may have been misclassified as cardia as the tumour margins may have expanded to that area of the stomach. Although our study did not find a significant association in individuals < 50 years of age, the point estimate was larger among younger individuals than in the older group. To our knowledge, this is the first study to evaluate the age-specific association between pernicious anaemia and gastric cancer. One piece of supporting evidence can be found in a prospective study reporting a significant association between anti-parietal cell antibody and gastric cancer risk among young females [33]. Assessment of subsite- and age-specific associations merits further investigation. Future studies should also examine associations with autoimmune conditions in different age categories within the sexes.

In this study, aplastic anaemia was nominally associated with gastric cancer, although they have not previously been linked directly. However, an association between aplastic anaemia and pernicious anaemia has been observed [24]. In our meta-analysis, significant positive pooled associations with gastric cancer were found for primary biliary cirrhosis, Graves’ disease and myasthenia gravis, but none of these associations was observed in our study.

Autoimmune conditions arise in a constellation where one individual can experience multiple such conditions simultaneously [28]. Thus, autoimmune conditions associated with pernicious anaemia may indirectly be linked to the risk of gastric cancer. In CPRD, we observed positive associations between pernicious anaemia and type 1 diabetes, hemolytic anaemia, Crohn’s disease, celiac disease, Sjögren’s disease, rheumatoid arthritis, and vitiligo, despite none of these conditions being significantly associated with gastric cancer. When we meta-analyzed these results with our recent SEER-Medicare study, Crohn’s disease, celiac disease, rheumatoid arthritis, ulcerative colitis, and polymyalgia rheumatica remained positively associated with pernicious anaemia [24]. Although no association was found in our study, including in our meta-analysis, another recent meta-analysis estimated that the relative risk of gastric cancer among people with type 1 diabetes was 46% greater than those without [34]. This observation may be evidence that pernicious anaemia mediates the association between other autoimmune conditions and gastric cancer.

## Strengths and limitations

The CPRD GOLD dataset’s large sample size is a strength that made our risk estimates precise and enabled us to conduct subgroup analyses. This database is also known for its high accuracy in cancer diagnoses [35]. The prospective design and the fact that information on exposures and diseases is collected from clinical records before any study hypothesis was developed also reduce the risk of recall bias [36]. Notably, we had a relatively large dataset that included young people ( < 50 years) who are prone to the onset of autoimmune diseases and often underrepresented in gastric cancer studies.

This study was limited by the lack of data on *H. pylori* infection and family history of gastric cancer. Furthermore, pernicious anaemia in the UK is often defined by vitamin B12 deficiency [37]. We did not have a variable for vitamin B12 deficiency or treatment and, therefore, only considered individuals to be exposed to pernicious anaemia if they had a specific medical provider code for pernicious anaemia, which may mean that we missed some people who truly had that autoimmune condition but only had a coded diagnosis of vitamin B12 deficiency. Nevertheless, we believe that our more strict definition of pernicious anaemia ensured that those considered exposed truly had that autoimmune condition. Additionally, a large proportion of BMI and smoking data were missing. However, BMI is not strongly associated with non-cardia gastric adenocarcinoma [38]. Our complete-case analysis results did not differ from our main results, suggesting that these covariates did not strongly bias the effect measure estimate. Finally, the prevalence of autoimmune conditions may have been underestimated in our dataset because people may be more likely to seek clinical care only after the onset of symptoms. Autoimmune conditions also tend to be diagnosed considerably earlier in life than gastric cancer, and the symptoms of such conditions can be managed or even put into remission at older ages.

## Conclusion

Our study and our large meta-analysis confirmed the positive association between autoimmune conditions and gastric cancer risk. Approximately 1-in-5 individuals with pernicious anaemia have at least one other autoimmune condition. Individuals with pernicious anaemia may benefit from gastric cancer screening. International consensus diagnostic criteria for autoimmune conditions and their co-occurrence are needed, as well as the establishment of large international registries linking individuals with autoimmune conditions to cancer incidence and mortality data, to improve our understanding of the effects of autoimmunity on cancer. In addition, further research is necessary to elucidate the biological mechanisms underlying the associations between autoimmune conditions and gastric cancer, with a particular focus on autoimmune gastritis as a potential pathway, while also considering other possible carcinogenic mechanisms.

### Supplementary information

Supplemental Material

## Data Availability

Access to data from CPRD is subject to a full license agreement containing detailed terms and conditions of use. Anonymised patient datasets can be extracted for researchers against specific study specifications, following protocol approval via CPRD’s Research Data Governance Process (https://cprd.com/data-access).
